# Distinct Innate and Adaptive Immune Modules Differentially Associate with HIV Reservoir Size and Decay During Early Antiretroviral Therapy

**DOI:** 10.3390/cells15131161

**Published:** 2026-06-25

**Authors:** Wei-Zhe Li, Hui-Huang Huang, Hui-Fang Wang, Xia Li, Ming-Ju Zhou, Yu-Xuan Yang, You-Yuan Wang, Meng-Meng Zhu, Ying Sun, Si-Yuan Chen, Xing Fan, Yan-Mei Jiao, Jin-Wen Song, Ruo-Nan Xu, Cheng Zhen, Ming Shi, Chao Zhang, Fu-Sheng Wang

**Affiliations:** 1 Medical School of Chinese People’s Liberation Army (PLA), Beijing 100853, China; lwzh0805@163.com (W.-Z.L.); youyuanwang197@163.com (Y.-Y.W.); 2 Senior Department of Infectious Diseases, Chinese PLA General Hospital, Beijing 100039, China; hhh302@163.com (H.-H.H.); lixia688lisa@163.com (X.L.); zhoumj89@163.com (M.-J.Z.); yangyuxuan0211@163.com (Y.-X.Y.); zhumengmeng@stu.pku.edu.cn (M.-M.Z.); 18254576112@163.com (Y.S.); chenzhuod@163.com (S.-Y.C.); fanxing302@aliyun.com (X.F.); jiaoyanmei@sina.com (Y.-M.J.); songjinwenchina@yeah.net (J.-W.S.); xuruonan2004@aliyun.com (R.-N.X.); zhencheng302@126.com (C.Z.); shiming302@sina.com (M.S.); 3 Department of Immunology & Microbiology, School of Basic Medical Sciences, Zhengzhou University, Zhengzhou 450001, China; hfw181331@zzu.edu.cn

**Keywords:** HIV reservoir, antiretroviral therapy (ART), innate immunity, adaptive immunity, NK cells, HIV-specific T cells

## Abstract

**Highlights:**

**What are the main findings?**

- HIV-1 DNA size and decay during the early period following ART initiation are immunologically distinct, governed by separate immune modules.
- Lower HIV-1 DNA size correlates with NK cell function, while faster decay is linked to restored HIV-specific T-cell responses.

**What are the implications of the main findings?**

- This work offers potential immune clues for exploring the viral reservoir and generates testable hypotheses for validation in future large cohorts.
- Targeting stage-specific immune modules may enable tailored HIV reservoir reduction strategies.

**Abstract:**

HIV reservoir size and decay represent distinct dimensions of viral persistence, yet whether they are governed by shared or separable immunological mechanisms during early antiretroviral therapy (ART) remains unclear. In this study, we employed multiparameter flow cytometry and bulk RNA sequencing to analyze longitudinal immune profiles across 21 treatment-naïve people living with HIV before ART initiation and at 1 and 5 months thereafter. Our findings revealed an apparent dissociation between HIV-1 DNA levels and decay rates in peripheral blood, and the two indicators appear to be relatively independent dimensions of viral persistence. Specifically, lower HIV-1 DNA levels were associated with higher frequencies of cytotoxic and adaptive-like natural killer (NK) cell subsets, whereas faster HIV-1 DNA decay was linked to restored HIV-specific CD4+ and CD8+ T-cell responses during treatment. Notably, transcriptomic analyses uncovered divergent gene expression signatures related to B cell-associated immunity and type I interferon pathways, with individuals with higher HIV-1 DNA levels exhibiting elevated expression of immunoglobulin and interferon-stimulated genes, while faster decay correlated with enrichment of antiviral and complement-related genes. Collectively, these findings provide a preliminary characterization of immune correlates of peripheral blood total HIV-1 DNA dynamics in the early phase following ART initiation. This work offers potential immune clues for exploring the viral reservoir and generates testable hypotheses for validation in future large cohorts.

## 1. Introduction

Globally, an estimated 40.8 million individuals are living with HIV, with 1.3 million new infections reported annually [1]. Despite the availability of effective antiretroviral therapy (ART) that achieves durable suppression of viral replication, HIV remains incurable [2]. The primary barrier to HIV cure lies in the latent viral reservoir, which is predominantly composed of quiescent memory CD4+ T cells harboring replication-competent proviruses [3]. This reservoir is seeded rapidly during the earliest stages of infection, becomes stably established within days to weeks, and subsequently exhibits heterogeneous and often biphasic decay kinetics following ART initiation [4].

Considerable efforts have been devoted to developing therapeutic strategies aimed at eliminating or functionally controlling the HIV reservoir. These include latency-reversing agents (LRAs), immune-based interventions such as immune checkpoint blockade (e.g., anti–PD-1 or anti–IL-10 antibodies), therapeutic vaccination, and gene-editing approaches including CRISPR–Cas systems [5,6,7,8]. However, these attempts are severely limited by non-selective cellular activation, inadequate immune clearance efficacy or off-target cytotoxicity, ultimately stemming from insufficient understanding of the viral reservoir status and its associated immunological factors [9].

The size and persistence of the HIV reservoir are tightly regulated by coordinated innate and adaptive immune responses. Innate immune mechanisms, including the cytolytic activity of natural killer (NK) cells, particularly adaptive or memory-like subsets that expand during acute infection and persist under suppressive ART, and type I interferon (IFN-I) signaling, exert complex and context-dependent effects on reservoir control, balancing early antiviral restriction against the induction of immune exhaustion and long-term reservoir stability [10,11,12,13,14,15]. In parallel, dendritic cells, especially lymph node–resident subsets, can harbor replication-competent HIV and facilitate viral persistence through antigen presentation and cell-to-cell transmission, underscoring the importance of tissue immune microenvironments in reservoir maintenance [16]. On the adaptive side, CD8+ T cells, including stem-like and CCL5-secreting virtual memory subsets, contribute to limiting reservoir persistence through cytolysis and cytokine secretion [15,17,18,19,20,21]. HIV-specific CD4+ T helper functions and B cell-derived neutralizing antibodies further contribute to immune-mediated control of the reservoir [9,18]. Despite these advances, most studies investigating immune–reservoir interactions have been cross-sectional or focused on isolated immune parameters, limiting their ability to capture dynamic changes or identify key determinants of reservoir decay in vivo.

Importantly, phylogenetic evidence suggests that while the latent HIV reservoir is seeded early in infection, the majority of the replication-competent, long-lived reservoir is established or stabilized around the time of ART initiation, rather than accumulating continuously throughout untreated chronic infection [22]. This renders the period around ART initiation a critical therapeutic window for the implementation of viral reservoir intervention strategies. A detailed, longitudinal characterization of reservoir dynamics during this phase, together with integrated immunophenotypic profiling, is therefore essential for identifying immunological correlates that may inform rational reservoir-targeting strategies.

In this study, we conducted a longitudinal analysis of total HIV-1 DNA levels and comprehensive immune phenotypes in ART-naive individuals initiating therapy. Our objective was to delineate early reservoir decay patterns following ART initiation and to identify innate and adaptive immune factors associated with reservoir size and decay kinetics, thereby providing insight into immune mechanisms that may be leveraged to improve HIV cure strategies.

## 2. Materials and Methods

### 2.1. Study Cohort

The longitudinal cohort of people living with HIV-1 (PLWH) was recruited at the Fifth Medical Center of Chinese PLA General Hospital from March 2021 to February 2023. Eligible participants were adults (≥18 years) with confirmed HIV-1 infection who were ART-naïve at enrollment and subsequently initiated standard antiretroviral therapy, with a minimum follow-up duration of five months. Individuals were excluded if they met any of the following criteria: prior ART exposure, pregnancy or lactation, drug resistance, co-infection with hepatitis B virus (HBV) or hepatitis C virus (HCV), active opportunistic infections, or comorbidities such as malignancies, severe organ dysfunction, or autoimmune diseases. Detailed clinical information is provided in Table 1.

### 2.2. HIV-1 DNA Detection and Decay Rate Analysis

Total cellular HIV-1 DNA was adopted as the surrogate marker for HIV reservoir assessment in this study, which is a widely applied approach in clinical cohort studies for evaluating viral reservoir burden and dynamics [23,24]. Total cellular HIV-1 DNA was extracted from isolated peripheral blood mononuclear cell (PBMC) samples using the QIAamp DNA Mini Kit (Qiagen, Valencia, CA, USA). DNA extracts were subsequently amplified and quantified with the HIV-1 total DNA quantitative detection kit (SUPBIO, Guangzhou, China) on the Roche LightCycler 480 real-time PCR system (Roche, Mannheim, Baden-Württemberg, Germany), as previously described [19]. HIV-1 DNA levels were expressed as copies per 1 × 10^6^ PBMCs.

To characterize reservoir decay dynamics after ART initiation, log10-transformed HIV-1 DNA measurements were fitted using individual-level linear regression models over time. The linear model was used uniformly across all subjects, and no special processing was performed for cases with non-linear decay trends. The decay rate was defined as the negative slope of the regression line, and the corresponding half-life was calculated assuming first-order decay kinetics [10].

### 2.3. Flow Cytometry

Cryopreserved peripheral blood mononuclear cells (PBMCs) were thawed, washed, and stained with the viability reagent Fixable Viability Stain 700 (BD Biosciences, San Jose, CA, USA, catalog #564997), fluorophore-conjugated antibodies, and Brilliant Stain Buffer Plus (BD Biosciences, San Jose, CA, USA, catalog #566385). Antibody panels for innate immune cell phenotyping, bulk T-cell profiling, and HIV-specific T-cell analysis are provided in Tables S1, S2 and S3, respectively. After surface staining, the cells were permeabilized and fixed using the eBioscience™ Foxp3 Permeabilization/Fixation Kit (Invitrogen, Carlsbad, CA, USA, catalog #00-5523-00) according to the manufacturer’s instructions. Then, cells were stained with antibodies to intracellular cytokines (Listed in Table S3). Data were acquired on a FACS Canto II flow cytometer (BD Biosciences, San Jose, CA, USA) and analyzed using FlowJo software v.10.8.1 (BD Biosciences, Ashland, OR, USA).

The frequency of HIV-specific CD4+ and CD8+ T cells was assessed by intracellular cytokine staining (ICS) following peptide stimulation. Prior to the aforementioned flow cytometric staining procedure, PBMCs from each participant were thawed and plated in 96-well plates at a density of 2 × 10^6^ cells/well in RPMI 1640 media (Thermo Fisher Scientific, Waltham, MA, USA, catalog #11875093) containing 10% fetal bovine serum (FBS, Thermo Fisher Scientific, Waltham, MA, USA, catalog #A5669201) and Penicillin/Streptomycin (Beyotime Biotechnology, Shanghai, China, catalog #C0222). Cells were stimulated with HIV peptide pools (1 μg of each peptide/mL, JPT Peptide Technologies, Berlin, Germany, catalog #PM-HIV-ENV/POL/GAG) in the presence of anti-CD28/CD49d (1 μL/mL, Biolegend, San Diego, CA, USA, catalog #377603/304339) and incubated at 37 °C for 6 h. Secretion inhibitors (Brefeldin A/Monensin, BD Biosciences, San Jose, CA, USA, catalog #555029/554724) were not included to avoid interference with AIM marker induction (e.g., CD69/4-1BB, Biolegend, San Diego, CA, USA).

### 2.4. Bulk RNA-Seq and Data Analysis

Total RNA was extracted from PBMCs using TRIzol^TM^ (Invitrogen, Carlsbad, CA, USA, catalog #15596018CN). The transcriptome sequencing was performed on the Illumina NovaSeq 6000 platform (Illumina, San Diego, CA, USA). To account for variations in sequencing depth and gene length, raw read counts were normalized to fragments per kilobase of transcript per million mapped reads (FPKM), ensuring cross-sample comparability for subsequent downstream analyses. Saturation analysis, inter-sample expression correlation analysis, and principal component analysis (PCA) were subsequently performed to assess potential batch effects (e.g., sequencing batches, sample processing timelines) on gene expression data.

Differential gene expression analysis was conducted using the edgeR v3.36.0 package implemented within the HIPLOT platform (https://hiplot.com.cn/home/index.html, accessed on 10 January 2026) and independently validated in R (version 4.5.2). To control for multiple hypothesis testing, *p* values were adjusted using the Benjamini–Hochberg false discovery rate (FDR) correction. Functional annotation and pathway enrichment analyses of DEGs were performed based on Gene Ontology (GO), Kyoto Encyclopedia of Genes and Genomes (KEGG), and Reactome databases to identify significantly enriched biological processes and signaling pathways. For pathway-level comparisons across samples, gene expression values were Z-score normalized, and the mean Z-score of all genes within each pathway was calculated to derive a composite pathway activity score.

### 2.5. Statistical Analyses

Cell proportions and absolute counts were presented as mean ± SEM. Non-parametric statistical tests were applied throughout owing to sample size and data distribution considerations. Differences between two groups were assessed with the Mann–Whitney U test for unpaired comparisons and the Wilcoxon signed-rank test for paired comparisons. Correlations between variables were evaluated using Spearman’s rank correlation test. A two-sided *p*-value < 0.05 was considered statistically significant. All correlation analyses adopted unadjusted *p*-values due to the exploratory study design. All statistical analyses and data visualization were conducted using R (version 4.5.2).

## 3. Results

### 3.1. Study Participants

**Figure 1:**
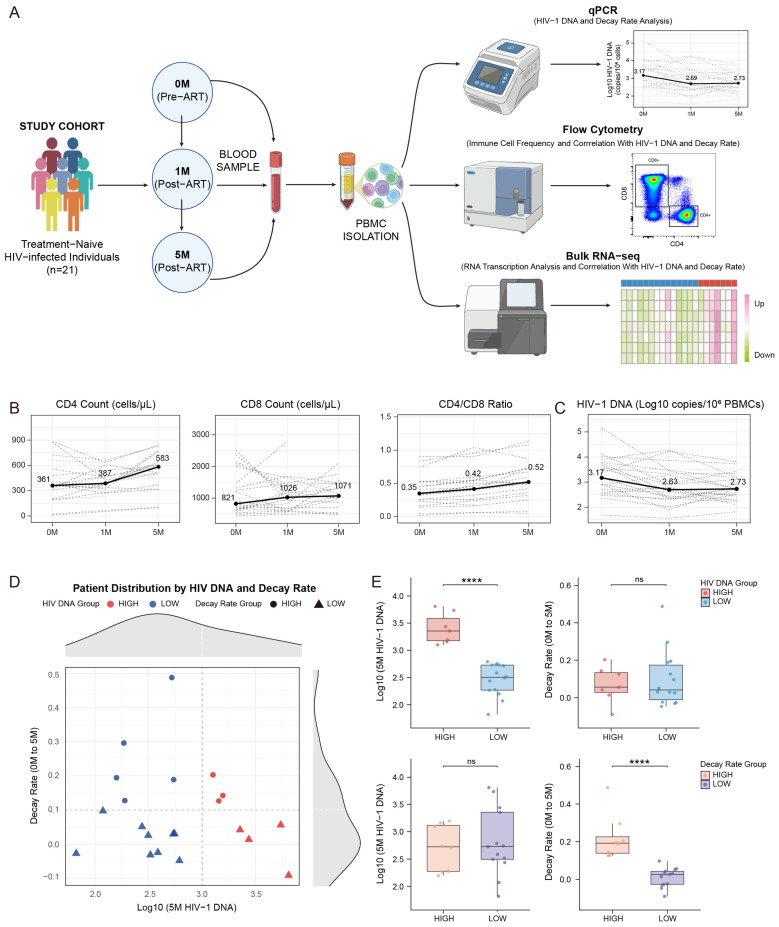
Independent Correlations Between HIV-1 DNA Size and Decay Rate During Early Antiretroviral Therapy (ART). (**A**) Schematic of the study design. 21 treatment-naïve PLWH were enrolled, with PBMC samples collected at 0M pre-ART, 1M, and 5M post-ART for HIV-1 DNA, flow cytometry, and bulk RNA-seq analyses. (**B**,**C**) Line graphs depict the dynamics of CD4+ and CD8+ T cell counts, CD4+/CD8+ ratio, and HIV-1 DNA in people living with HIV (PLWH) at baseline (0M), 1 month (1M), and 5 months (5M) after ART initiation. The solid black line represents the median value of each indicator across participants at each time point, with dashed lines showing individual participant trajectories. (**D**) Stratification of ART-treated individuals into HIGH/LOW HIV DNA groups and HIGH/LOW Decay Rate groups, based on HIV-1 DNA at 5M post-ART and decay rate (0M–5M). The cut-off value was set at 3 log10 copies per 10^6^ PBMCs for 5M HIV-1 DNA, and 0.1 log10 copies per 106 PBMCs per month for the decay rate (0M–5M). (**E**) Comparison of HIV-1 DNA levels at 5M post-ART and decay rates over 0M–5M between HIV DNA groups, and between Decay Rate groups. *p*-values were determined by the Mann–Whitney U test: **** *p* < 0.0001; ns, not significant (*p* ≥ 0.05).

A longitudinal cohort of 21 treatment-naïve people living with HIV (PLWH) was followed, with assessments conducted at three predefined time points: pre-ART (0M), 1 month (1M), and 5 months (5M) after ART initiation. The overall study design is illustrated in Figure 1A. All participants were male, with a median age of 31 years (range, 17–41 years). All cases were attributed to men who have sex with men (MSM) except for one patient with an unknown transmission route. To enhance sample heterogeneity, we enrolled participants with a broad range of pre-ART CD4+ T-cell counts, spanning from 11 to 888 cells/μL. The median pre-ART CD8+ T-cell count was 821 cells/μL (range, 436–2517 cells/μL), the median CD4/CD8 ratio was 0.35 (range, 0.02–0.90), and the median plasma viral load was 3.90 log10 copies/mL (range, 2.50–4.91 log10 copies/mL). Longitudinal changes in CD4+ T-cell count, CD8+ T-cell count, and CD4/CD8 ratio during ART are shown in Figure 1B. Plasma viral load dynamics are presented in Figure S1C, showing that all participants achieved viral suppression below the limit of detection by 5M post-ART. No residual viremia or viral blips were detected at the scheduled study visits.

### 3.2. HIV-1 DNA Size and Decay Rate as Independent Metrics During Early ART

To evaluate HIV-1 DNA size and decay rate during ART in study participants pre- and post-ART, we performed total HIV-1 DNA quantification based on peripheral blood mononuclear cells (PBMCs). Decay rates were calculated across three intervals (0M–1M, 0M–5M and 1M–5M) and subsequently converted to half-lives based on first-order decay kinetics [15]. The characteristics of HIV-1 DNA, decay rate, and half-life are summarized in Table 2. The dynamic changes in HIV-1 DNA during ART are depicted in Figure 1C. HIV-1 DNA levels exhibited a rapid decline from baseline (median, 3.17 log10 copies/10^6^ PBMCs) to 1M post-ART (median, 2.63 log10 copies/10^6^ PBMCs), followed by a plateau phase that persisted until 5M post-ART (median, 2.73 log10 copies/10^6^ PBMCs). Pre-ART CD4+ T-cell count was significantly negatively correlated with pre-ART HIV-1 DNA (r = −0.534, *p* = 0.013, Figure S1A). No significant correlations were observed between pre-ART CD8+ T-cell count, CD4/CD8 ratio, plasma viral load and HIV-1 DNA levels.

Given that HIV-1 DNA levels at 5M post-ART had entered a relatively stable plateau phase, we further plotted each individual based on 5M HIV-1 DNA and decay rate (0M–5M). As shown in Figure 1D, there was no consistent correlation between HIV-1 DNA size and decay rate: individuals with high decay rates were not necessarily accompanied by low HIV-1 DNA levels, and those with low decay rates did not inevitably exhibit high HIV-1 DNA levels. This finding suggests an observed dissociation between HIV-1 DNA size and decay kinetics, which may represent partially independent characteristics of the HIV reservoir.

Then, participants were stratified into two sets of subgroups: “HIGH HIV DNA” (*n* = 7) and “LOW HIV DNA” (*n* = 14) based on 5M HIV-1 DNA, and “HIGH Decay Rate” (*n* = 8) and “LOW Decay Rate” (*n* = 13) based on decay rate (0M–5M). The median HIV-1 DNA levels were 3.35 (range, 3.10–3.81) and 2.51 (range, 1.82–2.79) log10 copies per 10^6^ PBMCs for the HIGH and LOW HIV DNA groups, respectively. The decay rates did not differ significantly between these two groups (Figure 1E). The median decay rates for the HIGH and LOW Decay Rate groups were 0.192 (range, 0.126–0.488) and 0.026 (range, −0.089–0.097) log10 copies per 10^6^ PBMCs per month, respectively, with no significant difference in HIV-1 DNA levels between them (Figure 1E).

Next, we compared pre-ART clinical indicators across HIV DNA groups and Decay Rate groups (Tables S4 and S5). There were no significant differences in age or ART regimen between the two groups in either classification. Compared to the HIGH HIV DNA group, the LOW HIV DNA group had a significantly higher CD4+ T-cell count at baseline (*p* = 0.012), and this difference persisted at 1M and 5M post-ART (Figure S1B). This pattern is consistent with sustained immune impairment in individuals with larger HIV-1 DNA reservoirs. In contrast, CD8+ T-cell count, CD4/CD8 ratio, and plasma viral load were comparable between the two groups (Figure S1B). In summary, these results reveal an observed dissociation between HIV-1 DNA size and decay rate, two relatively independent metrics for evaluating the viral reservoir in the early period following ART initiation, likely governed by distinct immunological mechanisms.

### 3.3. Impact of Innate Immune Cell Subsets on HIV-1 DNA Size and Decay

**Figure 2:**
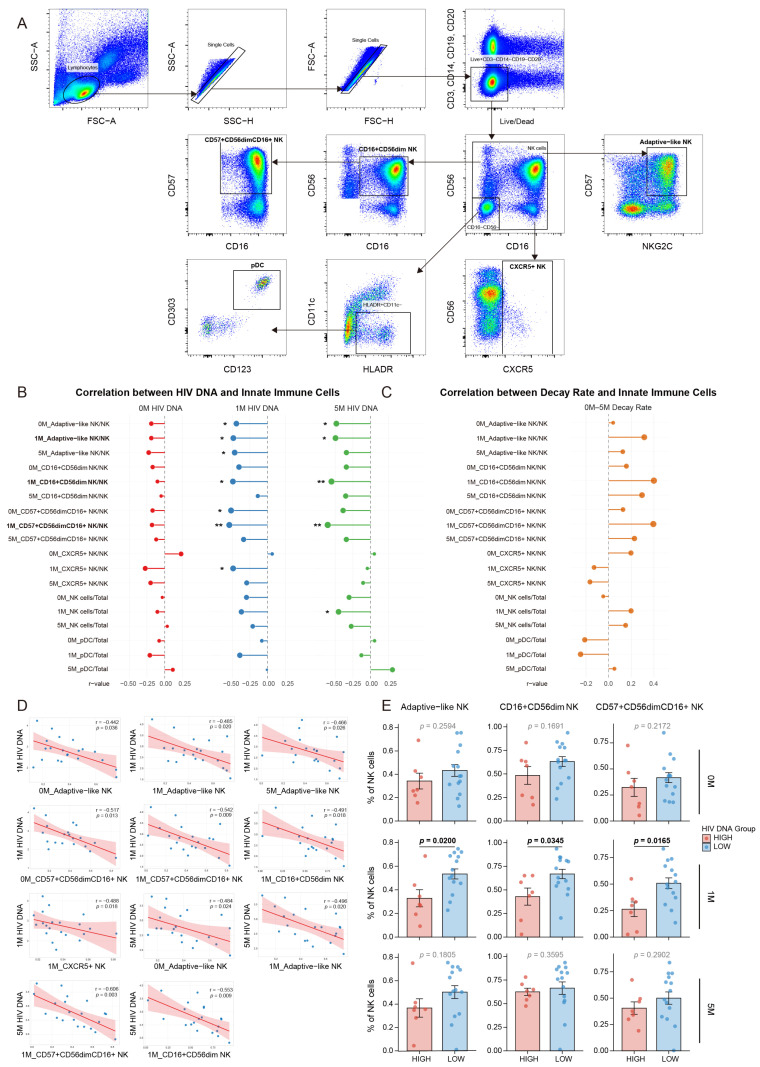
Association of Innate Immune Features with HIV-1 DNA Size and Decay. (**A**) Gating strategy for key innate immune cell subsets by flow cytometry. (**B**) Correlation lollipop plots illustrating the associations between innate immune cell subsets and HIV-1 DNA at 0M, 1M, and 5M. Each column represents HIV-1 DNA at distinct time points (color-coded). Each row denotes a specific innate immune cell variable across the three time points, sorted by immune cell type. Circle size corresponds to the magnitude of the correlation coefficient for each variable pair. Correlation coefficients were calculated using Spearman’s rank correlation method. Only significant correlations are shown (*p* < 0.05). * *p* < 0.05; ** *p* < 0.01. Bold labels highlight the cell subsets showing the most significant associations with HIV-1 DNA. (**C**) Correlations between innate immune cell subsets and HIV-1 DNA decay rates over the 5-month treatment course, sorted by immune cell type. (**D**) Scatter plots showing significant correlations between specific innate immune cell frequencies and HIV-1 DNA. (**E**) Comparisons of frequencies of innate immune cell subsets significantly associated with HIV-1 DNA between HIV DNA groups at 0M, 1M, and 5M. *p* values were determined by the Mann–Whitney U test.

Previous studies have shown that several innate immune cell subsets are associated with viral reservoir control in patients receiving long-term ART [10,11,12,13,14,15]. To better understand the role of innate immunity in the early period following ART initiation, we performed phenotypic analyses of plasmacytoid dendritic cells (pDCs) and NK cells in our study participants. Different functional subsets of NK cells were characterized based on the expression of CD16, CD56, CD57, NKG2C, and CXCR5 (Figure 2A). Correlation analyses revealed that several NK cell subsets were inversely associated with post-ART HIV-1 DNA levels. In particular, the frequencies of CD57+CD56dimCD16+ NK cells, CD16+CD56dim NK cells, and adaptive-like NK cells (NKG2C+CD57+) showed significant negative correlations with HIV-1 DNA levels at both 1M and 5M, with the strongest associations observed for measurements at 1M (Figure 2B,D). In contrast, no significant correlations were detected between frequencies of innate immune cell subsets at any time point and HIV-1 DNA decay rate (0M–5M) (Figure 2C).

Consistently, group-wise comparisons demonstrated that the LOW HIV DNA group exhibited significantly higher frequencies of CD57+ CD56dimCD16+ NK cells, CD16+ CD56dim NK cells, and adaptive-like NK cells at 1M compared with the HIGH HIV DNA group (*p* = 0.0165, *p* = 0.0345, and *p* = 0.02, respectively; Figure 2E). However, no differences were observed between HIGH and LOW Decay Rate groups (Figure S2). Together, these data suggest that specific NK cell subsets preferentially correlate with limiting HIV-1 DNA reservoir size rather than accelerating reservoir decay in the early period following ART initiation.

### 3.4. Impact of Adaptive Immune Cell Subsets on HIV DNA Size and Decay

Adaptive immunity is another crucial immunological factor involved in controlling the HIV reservoir [16,17,18,19,20,21]. To better understand the impact of host adaptive immunity on HIV DNA size and decay in the early period following ART initiation, we performed comprehensive phenotypic profiling of T-cell subsets in the study participants (Figure S3A), followed by correlation analyses with HIV-1 DNA size and decay rate. Consistent with absolute CD4+ T-cell counts, the pre-ART frequency of CD4+ T cells within PBMCs was negatively correlated with HIV-1 DNA (r = −0.47, *p* = 0.021). In contrast, the frequency of CD8+ T cells at 5M was positively correlated with baseline HIV-1 DNA (Figure S3B,D). Additionally, the frequency of CD28+CD4+ memory T cells at 5M showed a negative correlation with 1M HIV-1 DNA (Figure S3D). When examining decay kinetics, pre-ART CD4+ naïve T cells were negatively correlated with decay rate (0M–5M), whereas pre-ART CD28+CD4+ memory T cells exhibited a significant positive correlation (r = 0.45, *p* = 0.039, Figure S3C,D).

**Figure 3:**
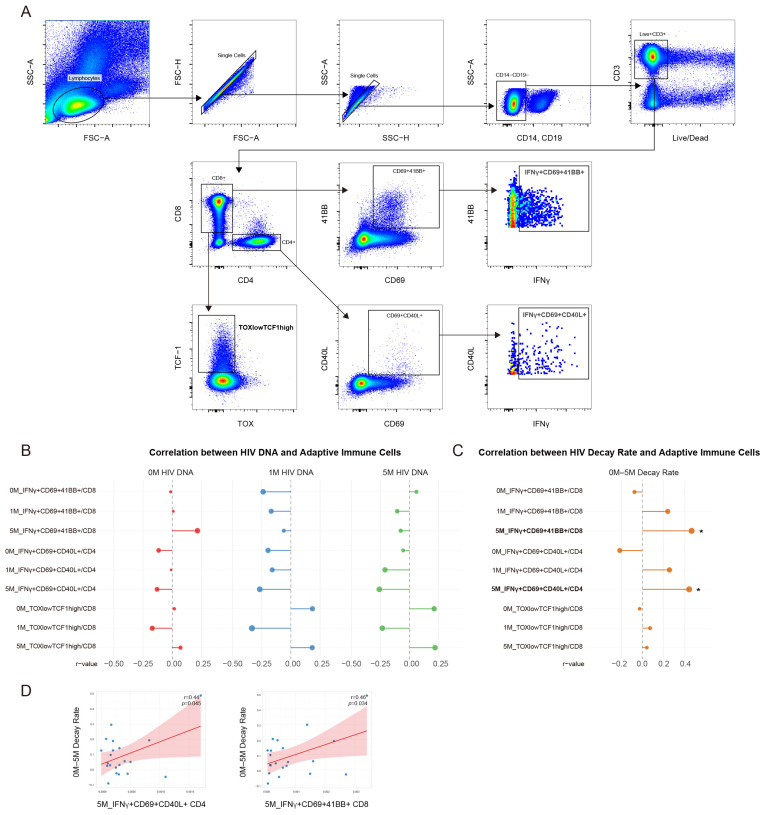
Association of Adaptive Immune Features with HIV-1 DNA Size and Decay. (**A**) Gating strategy for selected adaptive immune cells by flow cytometry. (**B**) Correlation lollipop plots illustrating the associations between selected adaptive immune cell subsets and HIV-1 DNA at 0M, 1M, and 5M. Each row denotes a specific adaptive immune cell variable across the three time points, sorted by immune cell type. Circle size corresponds to the magnitude of the correlation coefficient for each variable pair. Correlation coefficients were calculated using Spearman’s rank correlation method. Only significant correlations are shown (*p* < 0.05). * *p* < 0.05. Bold labels highlight the cell subsets showing the most significant associations with Decay Rate. (**C**) Correlations between selected adaptive immune cell subsets and HIV-1 DNA decay rates over the 5-month treatment course, sorted by immune cell type. (**D**) Correlation scatter plots showing adaptive immune cell subsets significantly associated with decay rate (0M–5M).

Next, we performed parallel analyses focusing on HIV-specific immune cell populations (Figure 3A). No significant correlations were observed between HIV-specific T cells and HIV-1 DNA at any of the three time points (Figure 3B). However, HIV-specific CD4+ and CD8+ T cell frequencies at 5M were both positively correlated with the decay rate during the 0M-5M period (r = 0.44, *p* = 0.045 and r = 0.46, *p* = 0.034, respectively; Figure 3C,D). These findings reveal correlative associations between adaptive immune parameters and faster decay of the HIV reservoir in the early period following ART initiation. We speculate that restored antigen-specific T cell responses may relate to the clearance of transcriptionally active or inducible reservoir cells rather than preventing initial reservoir seeding.

### 3.5. Distinct B Cell and Interferon Gene Signatures Underlying HIV DNA Size and Decay

**Figure 4:**
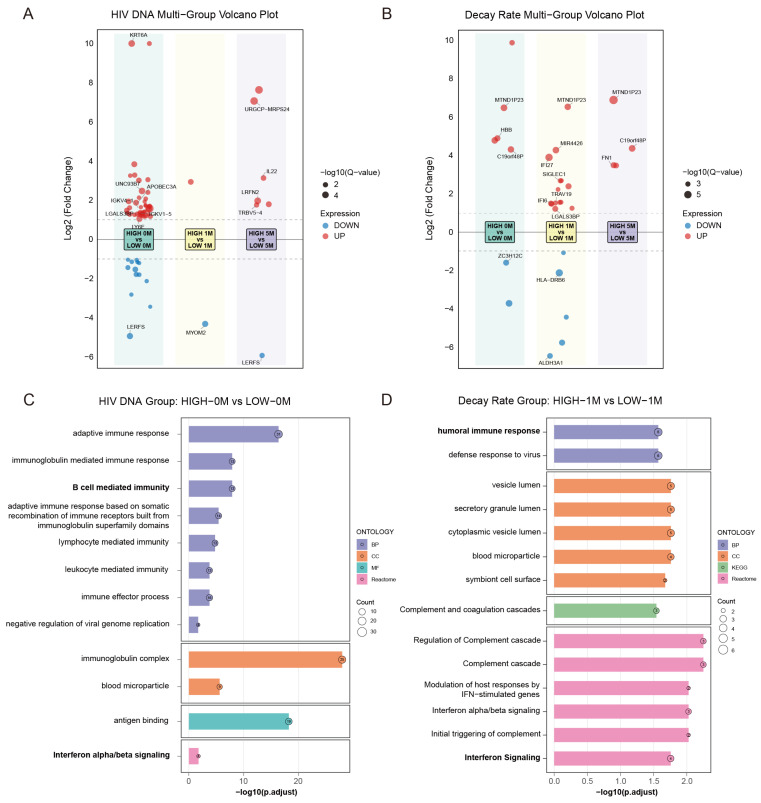
Bulk RNA-Seq and Functional Enrichment Analysis of HIV DNA and Decay Rate Groups. (**A**,**B**) Multi-group volcano plots displaying differentially expressed genes (DEGs) between HIV DNA groups and Decay Rate groups at 0M, 1M, and 5M. DEGs were filtered with thresholds of |log2 fold change| ≥ 1.0 and Q value < 0.05. Q values represent *p* values adjusted for multiple hypothesis testing. Dashed lines denote the cutoff of |log2 fold change| = 1.0. (**C**,**D**) GO, KEGG, and Reactome pathway enrichment analyses of DEGs identified in HIV DNA groups at 0M and Decay Rate groups at 1M. Bar color denotes the enrichment analysis method. Circle size and the number within indicate the count of genes enriched in the pathway. Bold labels denote enriched terms associated with both HIV-1 DNA and Decay Rate. Due to limited DEGs under stringent thresholds, enrichment criteria were relaxed to |log2 fold change| ≥ 1.0 and Q < 0.2 to facilitate robust biological pathway annotation.

To investigate molecular programs associated with HIV-1 DNA size and decay, bulk RNA sequencing was performed on longitudinal PBMC samples. Differentially expressed gene (DEG) analysis was conducted comparing HIV DNA groups and Decay Rate groups at each time point (Figure 4A,B). In HIGH vs. LOW HIV DNA groups, 40/13, 1/1 and 6/1 genes were up-/downregulated at 0M, 1M and 5M, respectively; in HIGH vs. LOW Decay Rate groups, the corresponding numbers were 5/2, 14/5, and 4/0. DEGs in HIV DNA groups were primarily identified at 0M, indicating that distinct immune statuses had already exerted an impact on HIV DNA size prior to ART initiation, whereas those in decay rate groups were mainly observed at 1M, implying that early immunological changes following ART initiation are linked to subsequent reservoir decay.

**Figure 5:**
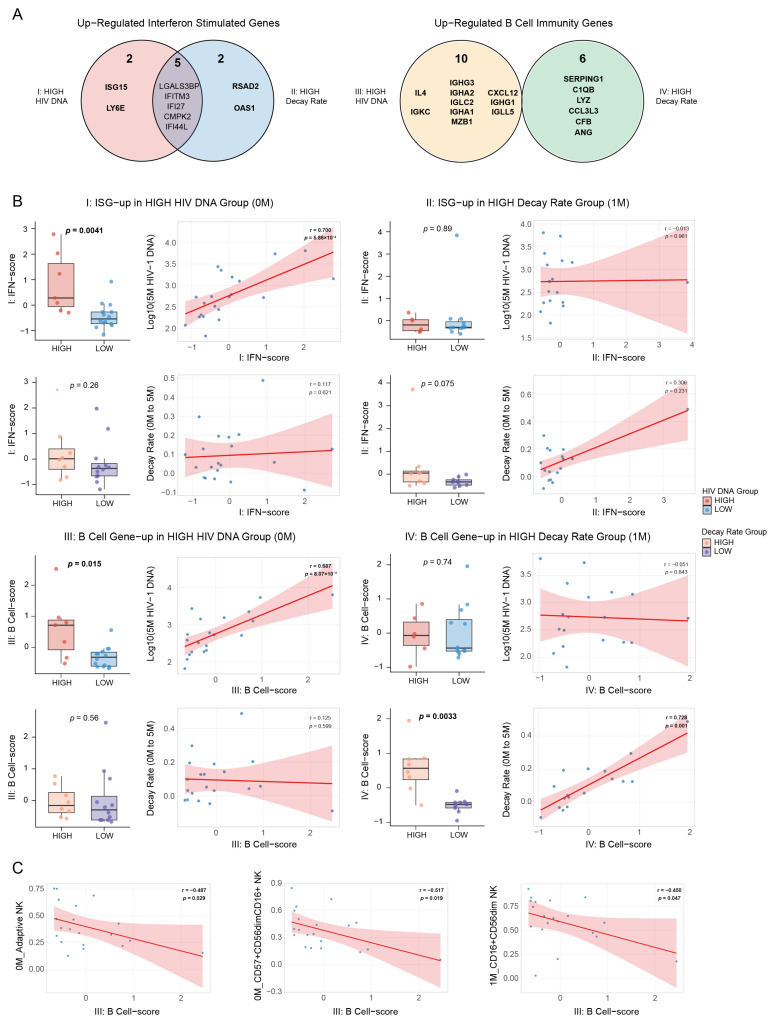
Distinct Gene Signatures in HIV DNA and Decay Rate Groups. (**A**) Venn diagrams illustrating the overlap of DEGs from type I interferon signaling and B cell-mediated immunity pathways between HIV DNA groups at 0M and between Decay Rate groups at 1M. (**B**) Analysis of four gene sets’ functional scores. Gene sets: I (ISGs—up in HIGH HIV DNA), II (ISGs—up in HIGH Decay Rate), III (B cell-mediated immunity genes—up in HIGH HIV DNA), IV (B cell-mediated immunity genes—up in HIGH Decay Rate). For each set, a composite Z-score was calculated per sample. Left panels: Comparison of Z-scores between HIV DNA groups and Decay Rate groups. *p*-values were determined by the Mann–Whitney U test. Right panels: Correlation of Z-scores with HIV-1 DNA at 5M and decay rate (0M–5M). Correlation coefficients were calculated using Spearman’s rank correlation method. (**C**) Significant correlations between specific innate immune cell frequencies and gene signature scores.

To further elucidate the functions of DEGs, we performed GO, KEGG and Reactome pathway enrichment analyses (Figure 4C,D). Enriched pathways included B cell-mediated immunity and type I interferon (IFN) signaling in HIV-1 DNA groups at 0M, and intriguingly, humoral immunity and type I IFN responses in Decay Rate groups at 1M. Despite overlap at the pathway level, gene-level signatures differed substantially between groups. Within the type I IFN signaling pathway, LGALS3BP, IFITM3, IFI27, CMPK2, and IFI44L were upregulated in both HIGH HIV DNA and HIGH Decay Rate groups, whereas ISG15 and LY6E were selectively upregulated in the HIGH HIV DNA group, and RSAD2 and OAS1 were specific to the HIGH Decay Rate group (Figures S4 and S5). Similarly, B cell-related genes upregulated in the HIGH HIV DNA group included IL4, IGKC, CXCL12, IGHG1, IGLL5, IGHG3, IGHA2, IGLC2, IGHA, and MZB1, whereas SERPING1, C1QB, LYZ, CCL3L3, CFB, and ANG were enriched in the HIGH Decay Rate group (Figure 5A, Figures S4 and S5).

To further quantify these pathway-level differences, four gene sets were defined and Z-score–normalized: Set I (interferon-stimulated genes (ISGs) enriched in HIGH HIV DNA), Set II (ISGs enriched in HIGH Decay Rate), Set III (B cell immunity genes enriched in HIGH HIV DNA), and Set IV (B cell immunity genes enriched in HIGH Decay Rate). Set I and Set III scores were significantly higher in the HIGH HIV DNA group and positively correlated with 5M HIV-1 DNA levels, but were unrelated to decay rate. In contrast, Set IV scores were significantly elevated in the HIGH Decay Rate group and positively correlated with decay rate (0M–5M), without association with HIV-1 DNA size (Figure 5B). Notably, Set III scores were inversely correlated with frequencies of 0M adaptive-like NK cells, CD57+CD56dimCD16+ NK cells and 1M CD16+CD56dim NK cells (Figure 5C). The causal relationship between B cell-related transcripts, NK cell subsets and HIV-1 DNA dynamics remains unclear and requires further mechanistic exploration.

## 4. Discussion

The latent HIV reservoir remains the principal barrier to viral eradication, and current therapeutic strategies targeting this reservoir are fundamentally constrained by an incomplete understanding of its regulatory mechanisms [9]. While both innate and adaptive immunity are known to participate in reservoir control, prior studies have largely been cross-sectional, focused on isolated factors, and have not sufficiently examined the critical early period during and immediately after ART initiation—when the reservoir is primarily stabilized and may be most amenable to intervention [22]. Through longitudinal analysis of 21 treatment-naïve PLWH, we show that HIV-1 DNA size and decay are distinct immunological dimensions, that innate and adaptive immunity constrain size and drive decay, respectively, and that both processes involve B cell and interferon pathways but divergent transcriptional programs. These findings offer new preliminary observations to support the development of targeted, stage-specific reservoir intervention strategies in future research.

A central observation of this study is that HIV-1 DNA levels at 5M post-ART do not correlate with decay rates measured over the same interval. This finding is consistent with recent work demonstrating that a high post-ART reservoir burden is unlikely to result from intrinsically slow decay kinetics [10]. Furthermore, a large-cohort prospective clinical study by Nadine Bachmann et al. revealed distinct clinical determinants underlying HIV DNA size and decay during prolonged suppressive ART [25]. While previous studies have predominantly evaluated reservoir size in relation to immunological parameters—often neglecting decay rate and failing to delineate distinct immunoregulatory mechanisms underlying these two metrics—our longitudinal analysis provides observational evidence for their partial dissociation. Mechanistically, reservoir size largely reflects the extent of early viral seeding and the state of immune suppression during initial infection, whereas decay rate is more closely tied to the efficiency of immune reconstitution following viral suppression [26,27]. This mechanistic divergence provides a biological rationale for evaluating reservoir size and decay rate as complementary, rather than interchangeable, endpoints in HIV cure research.

Our data further indicate that innate and adaptive immune responses contribute differentially to these two reservoir dimensions. NK cells, as key effectors of the innate immune system, play an important role in the immune response to HIV-1 infection [28]. We observed that multiple NK cell subsets, including CD57+CD56dimCD16+ NK cells, CD16+CD56dim NK cells, and adaptive-like NK cells, were inversely associated with post-ART HIV-1 DNA levels, consistent with previous reports [10,11,12,29]. In contrast, no association was detected between these NK subsets and reservoir decay rate. We speculate that NK cells preferentially target productively infected or recently activated cells, thereby limiting reservoir size, but lack the capacity to efficiently eliminate long-lived, transcriptionally silent CD4+ T cells that constitute the stable latent reservoir. This limitation underscores the need for combinatorial approaches to enhance NK cell-mediated clearance of latent reservoirs, such as dendritic cell-based activation strategies combined with immune checkpoint blockade (e.g., TIGIT inhibition) or HIV Env–CD16 bispecific antibodies, which have shown promise in preclinical models [28,30,31].

In contrast to innate immunity, adaptive immune responses, particularly HIV-specific T cells, were associated with reservoir decay rather than size. CD8+ cytotoxic T lymphocytes (CTLs) play a central role in controlling HIV replication [32,33], but chronic antigen exposure leads to functional exhaustion of these cells. Importantly, suppressive ART can partially restore HIV-specific T-cell function [18,32,33,34,35]. In our cohort, HIV-specific CD4+ and CD8+ T-cell responses correlated positively with reservoir decay rates but showed no association with total HIV-1 DNA levels. These findings are consistent with the conclusions of existing studies and further support the rationale for exploring therapeutic strategies (e.g., latency-reversing agents and vaccines) to modulate the function of HIV-specific CD8+ T cells for viral reservoir reduction [36,37,38,39,40]. Collectively, our findings reveal correlative links between immune cells and reservoir dynamics, providing a rationale for developing potential strategies to complement antiretroviral treatment for HIV-1 cure.

Transcriptomic analyses further reinforced the conceptual separation between reservoir size and decay. Differentially expressed genes associated with reservoir size were predominantly detected at baseline, whereas those linked to decay rate emerged mainly at 1M post-ART, suggesting a temporal hierarchy in molecular regulation. Although both processes involved B cell-mediated immunity and type I interferon signaling pathways, the underlying gene signatures differed markedly. The HIGH HIV DNA group exhibited elevated baseline expression of several type I interferon-related genes (e.g., LY6E, LGALS3BP) and immunoglobulin heavy-chain genes, which have been implicated in enhanced viral entry, antibody-dependent enhancement (ADE), or immune activation, potentially facilitating reservoir expansion [41,42]. In contrast, the HIGH decay rate group displayed increased post-ART expression of antiviral IFN-stimulated genes (e.g., OAS1, RSAD2) and complement-related genes (e.g., SERPING1, C1QB), consistent with enhanced viral RNA degradation and humoral effector function [43,44]. These data suggest that qualitatively distinct IFN and B cell programs may either stabilize or erode the reservoir, depending on timing and functional orientation. Bulk RNA-seq data may be confounded by changes in cellular composition, and DEGs were identified using a relaxed Q < 0.2 threshold. Notably, bulk RNA-seq results are susceptible to confounding effects from altered cellular composition during immune reconstitution, and differentially expressed genes were screened using a lenient Q < 0.2 threshold. For these reasons, the above transcriptomic findings require further validation in future studies.

This study has several important limitations that should be acknowledged. First, the total sample size was small (n = 21), and subgroup stratification further lowered statistical power. Correlation analyses used unadjusted *p*-values without multiple-comparison correction, and a relaxed Q < 0.2 threshold was used for DEG identification, raising false-positive risks [45,46]. Second, we measured total HIV-1 DNA in PBMCs to assess the viral reservoir, which fails to distinguish defective and intact proviruses and cannot represent tissue-resident reservoirs [47,48,49]. Third, we only analyzed immune cell phenotypes and did not conduct relevant functional assays, so the observed associations cannot be explained at the functional level. Bulk RNA-seq data are also susceptible to confounding effects caused by dynamic changes in PBMC composition during immune reconstitution after ART [50]. Collectively, all results from this exploratory study require further validation in larger, independent cohorts.

In summary, this study provides a longitudinal analysis of immunological and transcriptomic correlates of HIV reservoir dynamics in the early period following ART initiation. Our findings support the notion that reservoir size and decay rate reflect partially distinct biological processes, are differentially associated with innate and adaptive immune responses, and are accompanied by divergent yet overlapping molecular signatures. Together, these results put forward testable hypotheses and may help inform the design of future studies and therapeutic strategies aimed at promoting HIV reservoir reduction.

## Figures and Tables

**Table 1:** Characteristics of Study Participants.

Characteristic	Value
Sex, No. (%)	
Male	21 (100)
Female	0 (0)
Age, y	
Median (IQR)	31 (25–34)
Range	18–41
Time of untreated HIV-1 infection in years, No. (%)	
<1	20 (95.2)
>1	1 (4.8)
Transmission group by sex, No. (%)	
MSM	20 (95.2)
Unknown	1 (4.8)
Pre-ART CD4+ T cell count, cells/μL	
Median (IQR)	361 (200–479)
Range	11–888
Pre-ART CD8+ T cell count, cells/μL	
Median (IQR)	821 (624–1460)
Range	436–2517
Pre-ART CD4+/CD8+ T cell count ratio	
Median (IQR)	0.35 (0.25–0.52)
Range	0.02–0.90
Pre-ART plasma viremia, log10 copies/mL	
Median (IQR)	3.90 (3.41–4.43)
Range	2.50–4.91
ART regimens, No. (%)	
2 NRTIs + 1 INSTIs	3 (14.3)
2 NRTIs + 1 NNRTIs	14 (66.7)
2 NRTIs + 1 PIs	4 (19.0)

IQR, interquartile range; ART, antiretroviral therapy; NRTIs, nucleoside reverse transcriptase inhibitors; INSTIs, integrase strand transfer inhibitors; NNRTIs, non-nucleoside reverse transcriptase inhibitors; PIs, protease inhibitors.

**Table 2:** Characteristics of HIV-1 DNA.

Characteristic	Value
HIV DNA, log10 copies/10^6^ PBMCs	
0M	3.17 (2.63–3.75)
1M	2.63 (2.35–3.10)
5M	2.73 (2.44–3.16)
Decay Rate	
0M–1M	0.26 (0.07–0.90)
0M–5M	0.05 (0.01–0.14)
1M–5M	0.03 (−0.08–0.09)
Half-life, days	
0M–1M	44 (20–110)
0M–5M	189 (109–533)
1M–5M	244 (163–510)

## Data Availability

The original contributions presented in this study are included in the article and Supplementary Materials. Further inquiries can be directed to the corresponding authors.

## Supplementary Materials

The following supporting information can be downloaded at https://www.mdpi.com/article/10.3390/cells15131161/s1: Figure S1: Comparison of Clinical Parameters Between HIV DNA Groups and Decay Rate Groups. (A) Correlation between pre-ART (0M) CD4+ T cell count and HIV-1 DNA at 0M, 1M, and 5M. Correlation coefficients were calculated using Spearman’s rank correlation method. (B) Comparison of absolute CD4+/CD8+ T cell counts and CD4+/CD8+ ratio at baseline, 1M, and 5M between HIV DNA groups, and between Decay Rate groups. *p*-values were determined by the Mann–Whitney U test: * *p* < 0.05; ** *p* < 0.01; ns, not significant (*p* ≥ 0.05). (C) The dynamic changes in plasma viral load during the treatment period. LD, limit of detection; Figure S2: Comparisons of frequencies of innate immune cell subsets significantly associated with HIV-1 DNA between Decay Rate groups at 0M, 1M, and 5M. *p* values were determined by the Mann–Whitney U test; Figure S3: Association of Adaptive Immune Features with HIV-1 DNA Reservoir Size and Decay. (A) Gating strategy for selected adaptive immune cell subsets. (B) Correlation lollipop plots illustrating the associations between selected adaptive immune cell subsets and HIV-1 DNA at 0M, 1M, and 5M. Presentation details are as in Figure S3B. Only significant correlations (*p* < 0.05) are shown. * *p* < 0.05; ** *p* < 0.01. (C) Correlations between selected adaptive immune cell subsets and HIV-1 DNA decay rates over the 5-month treatment course, sorted by immune cell type. (D) Scatter plots showing significant correlations between specific adaptive immune cell frequencies and HIV-1 DNA (5M) or decay rate (0M–5M); Figure S4: Differential Expression Analysis of Type I Interferon Signaling and B Cell-Mediated Immunity Pathways. (A,B) Volcano plots of DEGs involved in type I interferon signaling and B cell-mediated immunity pathways between HIV DNA groups at 0M and between Decay Rate groups at 1M. (C,D) Heatmaps showing expression levels of DEGs from these pathways in the respective group comparisons; Figure S5: Comparison of expression levels of DEGs in type I interferon signaling and B cell-mediated immunity pathways between HIV DNA groups at 0M and between Decay Rate groups at 1M. *p*-values were determined by the Mann–Whitney U test. Table S1: Panel of antibodies used for innate immune cell phenotyping. Table S2: Panel of antibodies used for T cell phenotyping. Table S3: Panel of antibodies used for HIV-specific T cell phenotyping. Table S4: Pre-ART clinical indicators between HIV DNA groups. Table S5: Pre-ART clinical indicators between Decay Rate groups.

## Abbreviations

The following abbreviations are used in this manuscript:

ART	Antiretroviral therapy
PLWH	People living with HIV
HIV-1 DNA	Human immunodeficiency virus type 1 DNA
PBMCs	Peripheral blood mononuclear cells
NK cells	Natural killer cells
pDCs	Plasmacytoid dendritic cells
CTLs	Cytotoxic T lymphocytes
DEGs	Differentially expressed genes
FPKM	Fragments per kilobase transcript per million mapped reads
PCA	Principal component analysis
FDR	False discovery rate
GO	Gene Ontology
KEGG	Kyoto Encyclopedia of Genes and Genomes
ISGs	Interferon-stimulated genes
IFN-I	Type I interferon
ICS	Intracellular cytokine staining
FACS	Fluorescence-activated cell sorting
HBV	Hepatitis B virus
HCV	Hepatitis C virus
MSM	Men who have sex with men
LRAs	Latency-reversing agents
ADE	Antibody-dependent enhancement
