# Supplementary material for: Distinct Innate and Adaptive Immune Modules Differentially Associate with HIV Reservoir Size and Decay During Early Antiretroviral Therapy
Source: Cells. 2026 Jun 25;15(13):1161. doi: 10.3390/cells15131161 (PMC13359910; doi:10.3390/cells15131161)
Supplement: Supplementary file 1 [file cells-15-01161-s001.zip › cells-4320351-supplementary.pdf]

Figure S1

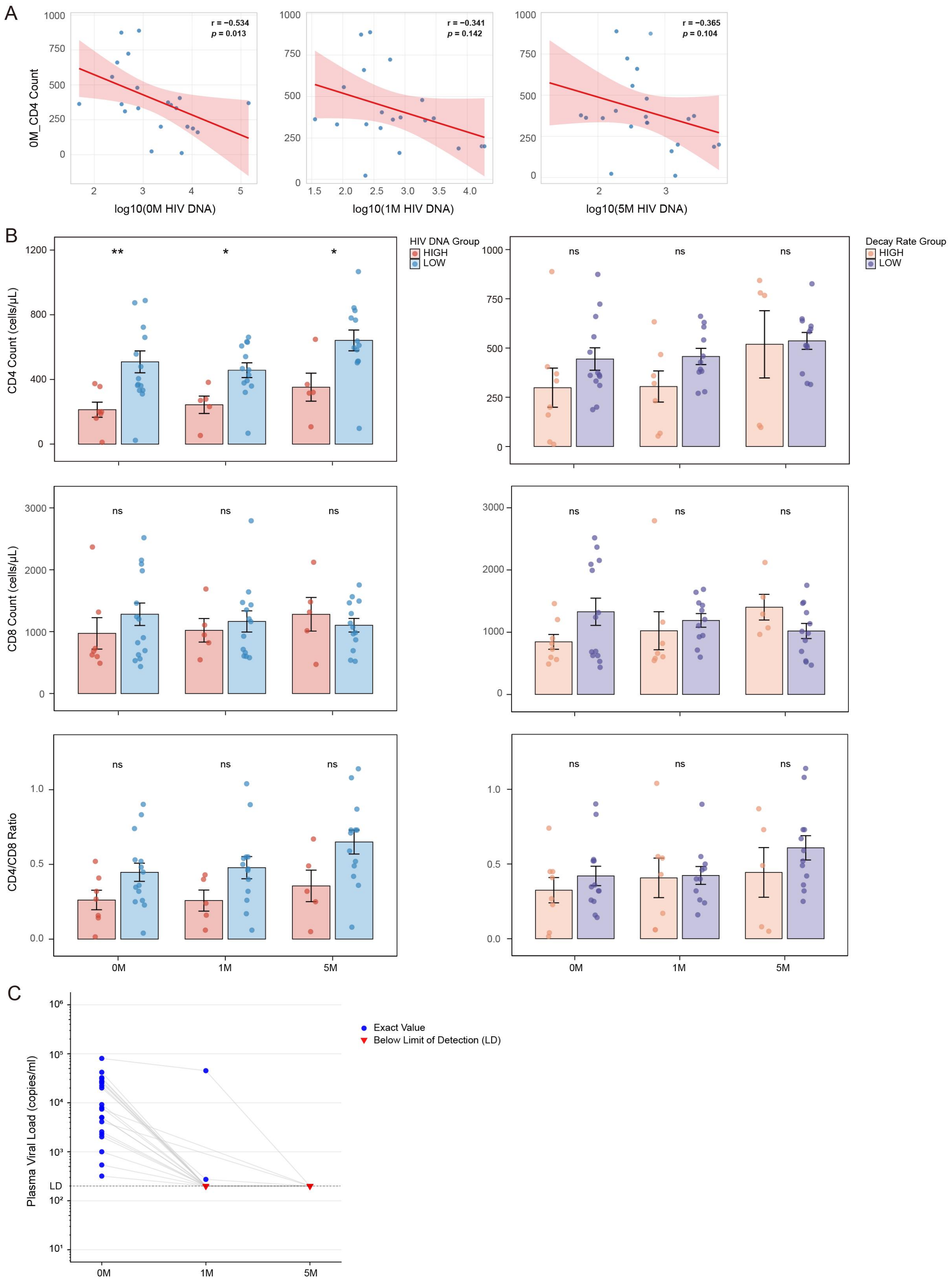
A
r = −0.534
p = 0.013
r = −0.341
p = 0.142
r = −0.365
p = 0.104
0M_CD4 Count
log10(0M HIV DNA)
log10(1M HIV DNA)
log10(5M HIV DNA)
B
HIV DNA Group
HIGH
LOW
Decay Rate Group
HIGH
LOW
CD4 Count (cells/μL)
CD8 Count (cells/μL)
CD4/CD8 Ratio
**
*
*
ns
0M
1M
5M
C
Exact Value
Below Limit of Detection (LD)
Plasma Viral Load (copies/ml)
LD

Figure S2

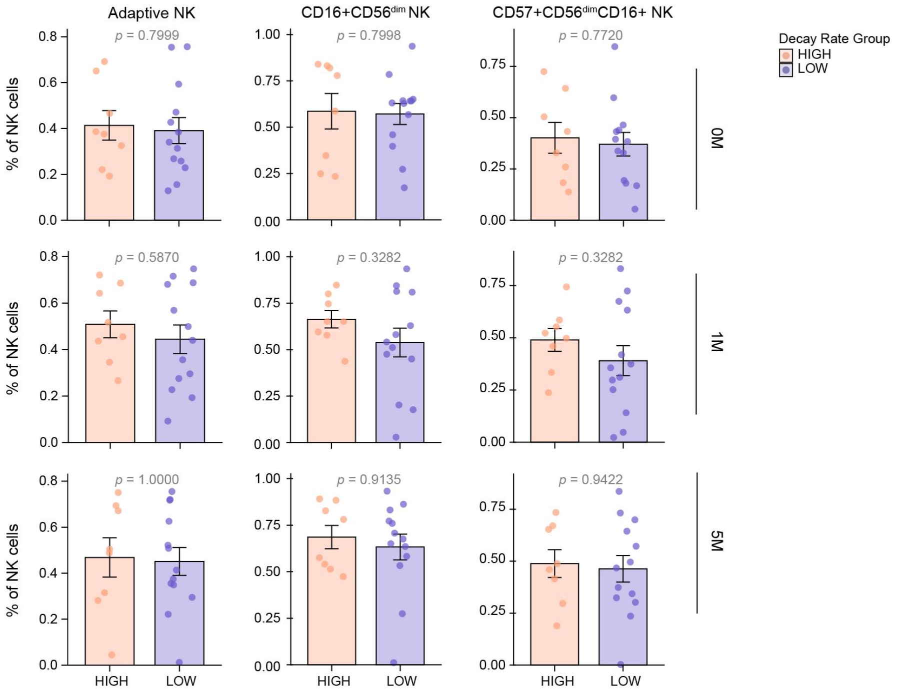

Adaptive NK
CD16+CD56dim NK
CD57+CD56dimCD16+ NK
Decay Rate Group
HIGH
LOW
% of NK cells
p = 0.7999
p = 0.7998
p = 0.7720
p = 0.5870
p = 0.3282
p = 0.3282
p = 1.0000
p = 0.9135
p = 0.9422
0M
1M
5M
HIGH
LOW

Figure S3

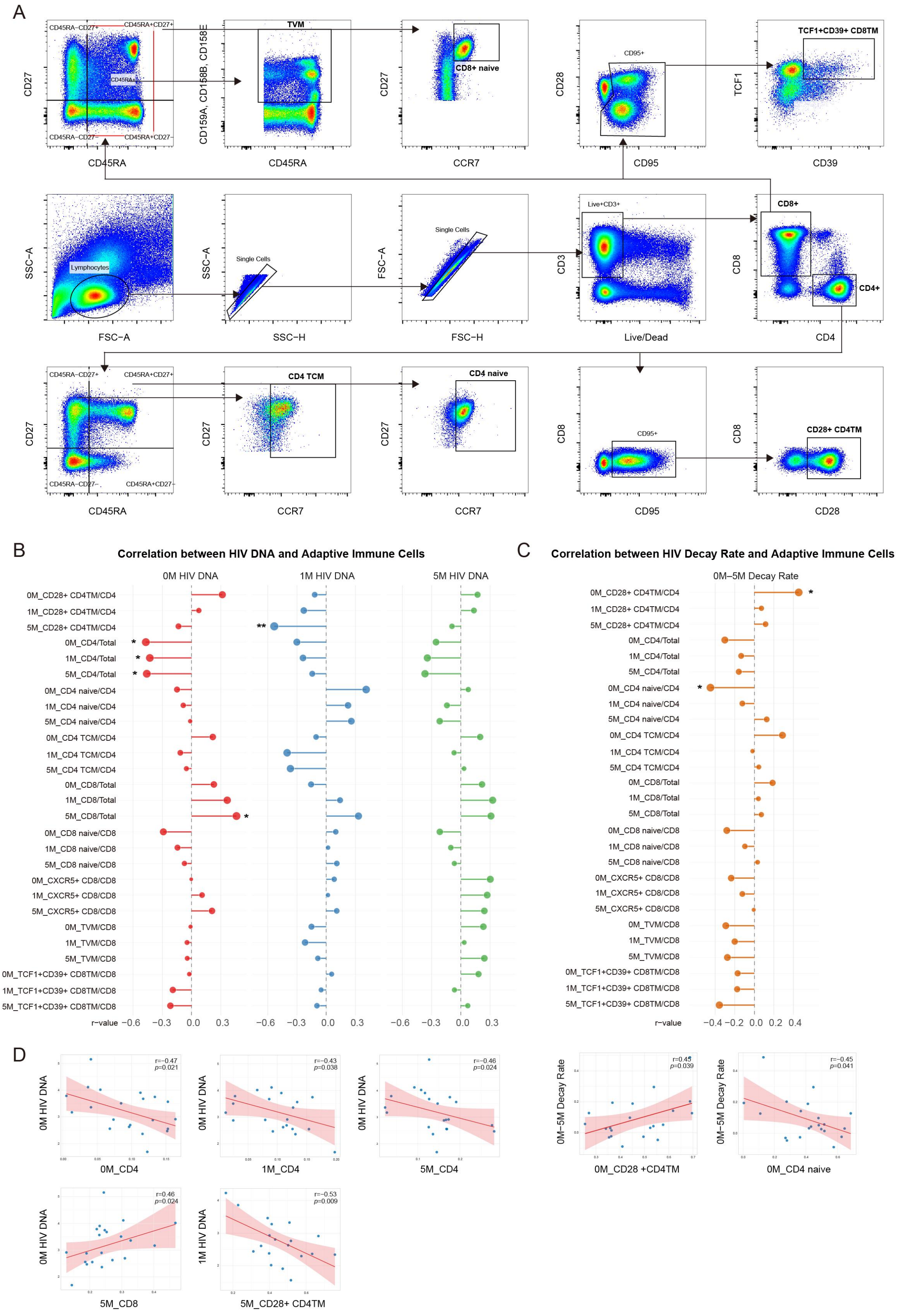
A
B
Correlation between HIV DNA and Adaptive Immune Cells
0M HIV DNA
1M HIV DNA
5M HIV DNA
C
Correlation between HIV Decay Rate and Adaptive Immune Cells
0M–5M Decay Rate
D

Figure S4

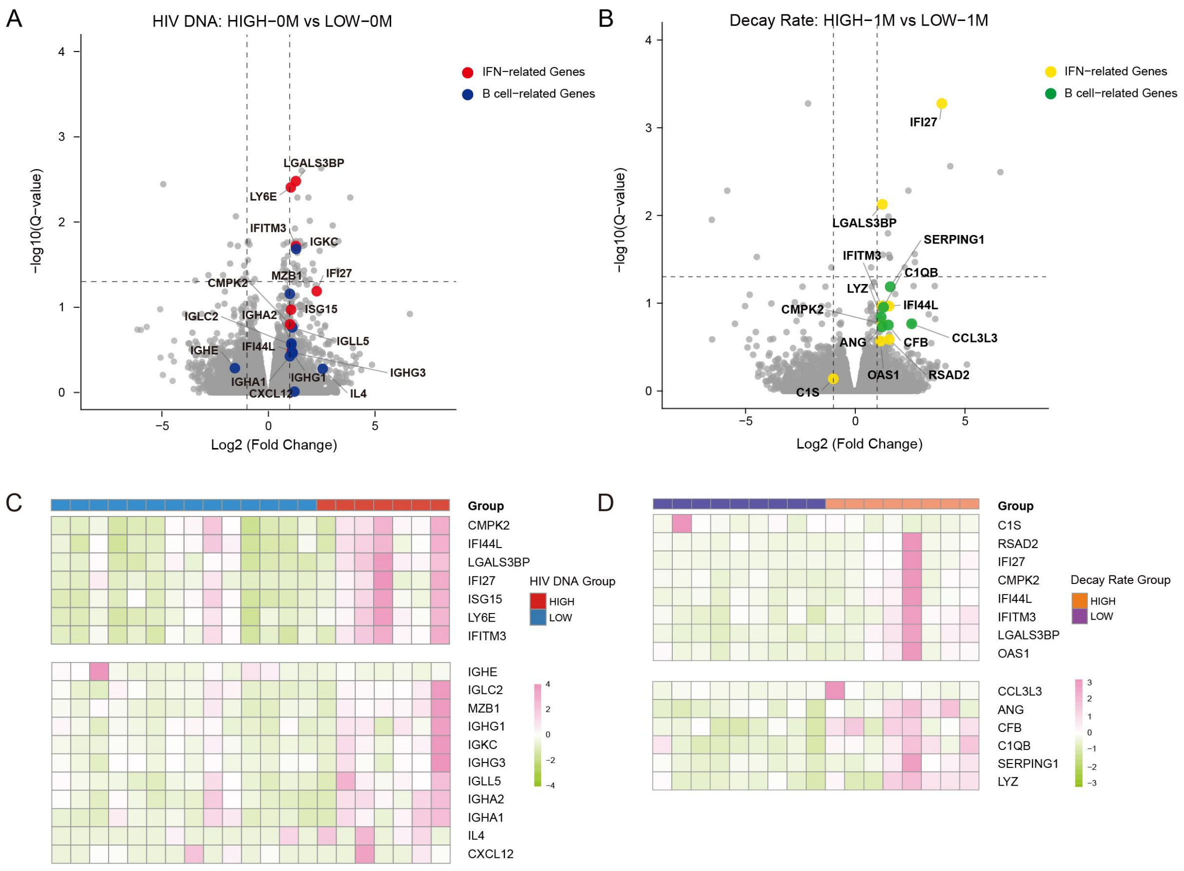
A
HIV DNA: HIGH−0M vs LOW−0M
IFN−related Genes
B cell−related Genes
−log10(Q−value)
Log2 (Fold Change)
LGALS3BP
LY6E
IFITM3
IGKC
MZB1
IFI27
CMPK2
ISG15
IGLC2
IGHA2
IGLL5
IGHE
IFI44L
IGHG3
IGHA1
IGHG1
CXCL12
IL4
B
Decay Rate: HIGH−1M vs LOW−1M
IFN−related Genes
B cell−related Genes
IFI27
LGALS3BP
SERPING1
IFITM3
C1QB
LYZ
IFI44L
CMPK2
CCL3L3
ANG
CFB
OAS1
RSAD2
C1S
C
Group
CMPK2
IFI44L
LGALS3BP
IFI27
ISG15
LY6E
IFITM3
HIV DNA Group
HIGH
LOW
IGHE
IGLC2
MZB1
IGHG1
IGKC
IGHG3
IGLL5
IGHA2
IGHA1
IL4
CXCL12
D
Group
C1S
RSAD2
IFI27
CMPK2
IFI44L
IFITM3
LGALS3BP
OAS1
Decay Rate Group
HIGH
LOW
CCL3L3
ANG
CFB
C1QB
SERPING1
LYZ

Figure S5

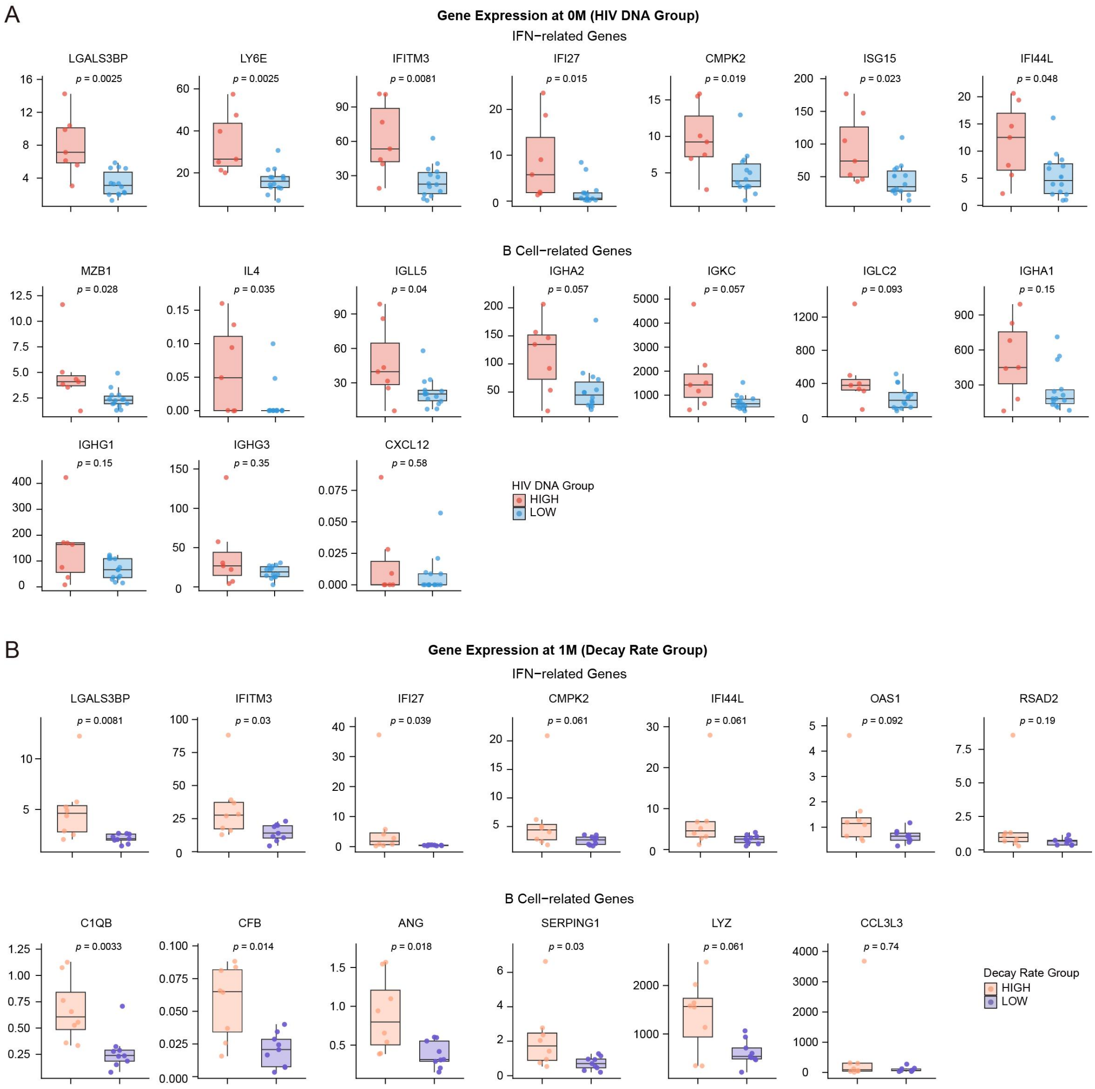
A
Gene Expression at 0M (HIV DNA Group)
IFN−related Genes
LGALS3BP p = 0.0025
LY6E p = 0.0025
IFITM3 p = 0.0081
IFI27 p = 0.015
CMPK2 p = 0.019
ISG15 p = 0.023
IFI44L p = 0.048
B Cell−related Genes
MZB1 p = 0.028
IL4 p = 0.035
IGLL5 p = 0.04
IGHA2 p = 0.057
IGKC p = 0.057
IGLC2 p = 0.093
IGHA1 p = 0.15
IGHG1 p = 0.15
IGHG3 p = 0.35
CXCL12 p = 0.58
HIV DNA Group
HIGH
LOW
B
Gene Expression at 1M (Decay Rate Group)
IFN−related Genes
LGALS3BP p = 0.0081
IFITM3 p = 0.03
IFI27 p = 0.039
CMPK2 p = 0.061
IFI44L p = 0.061
OAS1 p = 0.092
RSAD2 p = 0.19
B Cell−related Genes
C1QB p = 0.0033
CFB p = 0.014
ANG p = 0.018
SERPING1 p = 0.03
LYZ p = 0.061
CCL3L3 p = 0.74
Decay Rate Group
HIGH
LOW

Table S1. Panel of antibodies used for innate immune cell phenotyping

Antibody	Clone	Catalog Number	Company
CD3-BV510	HIT3A	564713	BD Biosciences
CD14-BV510	M5E2	301841	Biologend
CD19-BV510	HIB19	302241	Biologend
CD20-BV510	2H7	302339	Biologend
CD16-BV786	3G8	563689	BD Biosciences
CD56-BV421	5.1H11	362552	Biologend
CD57-BV711	QA17A04	393328	Biologend
CXCR5-PE-CF594	J252D4	356927	Biologend
CD123-BV605	6H6	306025	Biologend
CD303-PE	201A	354203	Biologend
CD11c-PE-CY7	3.9	301607	Biologend
HLA-DR-AF488	L243	307619	Biologend
FcεRI-FITC	N/A	FCABS400F	MERCK
NKG2C-BUV737	134591	749685	BD Biosciences

N/A = Not Applicable

Table S2. Panel of antibodies used for T cell phenotyping

Antibody	Clone	Catalog Number	Company
CD3-BUV737	SK7	612753	BD Biosciences
CD4-BUV496	SK3	612937	BD Biosciences
CD8-BUV395	RPA-T8	563795	BD Biosciences
CD45RA-BV785	HI100	304139	Biologend
CCR7-BV510	G043H7	353231	Biologend
CD27-BV605	O323	302829	Biologend
CD28-BV711	CD28.2	302948	Biologend
CD159a (NKG2A)-PE	REA110	130-113-566	Miltenyi Biotec
CD158b/j (KIR2DL2/DL3)-PE	DX27	312605	Biologend
CD158e (KIR3DL1)-PE	DX9	130-092-473	Miltenyi Biotec
CD95-PE-CY7	DX2	305622	Biologend
CD39-BB515	TU66	565469	BD Biosciences
Tox-APC	TXRX10	50-6502-82	eBioscience
TCF1-BV421	S33-966	566692	BD Biosciences
CXCR5-PE/Dazzle™ 594	J252D4	356927	Biologend

Table S3. Panel of antibodies used for HIV-specific T cell phenotyping

Antibody	Clone	Catalog Number	Company
CD14-BV510	M5E2	301841	Biologend
CD19-BV510	HIB19	302241	Biologend
CD3-BUV737	SK7	612753	BD Biosciences
CD4-BUV496	SK3	612937	BD Biosciences
CD8-BUV395	RPA-T8	563795	BD Biosciences
CD69-FITC	FN50	310903	BioLegend
CD40L-BV711	24-31	310838	BioLegend
4-1BB-PE	4B4-1	309803	BioLegend
IFN-γ-PE-CY7	4S.B3	502527	BioLegend
Tox-APC	TXRX10	50-6502-82	eBioscience
TCF1-BV421	S33-966	566692	BD Biosciences

Table S4. Pre-ART clinical indicators between HIV DNA groups

Characteristic	HIGH HIV DNA (n=7)	LOW HIV DNA (n=14)	*P* value
Age, y			
Median (IQR)	32 (28-35)	28 (23-33)	0.477
Range	19-40	17-41	
Pre-ART CD4+ T-cell count, cells/μL			
Median (IQR)	200 (174-278)	387 (340-634)	**0.012**
Range	11-374	23-888	
Pre-ART CD8+ T-cell count, cells/μL			
Median (IQR)	684 (611-1022)	1053 (643-1861)	0.433
Range	489-2367	436-2517	
Pre-ART CD4+/CD8+ T-cell count ratio			
Median (IQR)	0.27 (0.15-0.36)	0.40 (0.27-0.53)	0.101
Range	0.02-0.52	0.040-0.90	
Pre-ART plasma viremia, log10 copies/mL			
Median (IQR)	3.87 (3.66-4.26)	4.13 (3.33-4.42)	0.682
Range	3.41-4.91	2.50-4.51	
ART regimens, %			
2 NRTIs+1 INSTIs	2 (28.6)	1 (7.1)	0.251
2 NRTIs+1 NNRTIs	3 (42.9)	11 (78.6)	
2 NRTIs+1 PIs	2 (28.6)	2 (14.3)	

IQR, interquartile range; ART, antiretroviral therapy; NRTIs, nucleoside reverse transcriptase inhibitors; INSTIs, integrase strand transfer inhibitors; NNRTIs, non-nucleoside reverse transcriptase inhibitors; PIs, protease inhibitors.

Table S5. Pre-ART clinical indicators between Decay Rate groups

Characteristic	HIGH Decay Rate (n=8)	LOW Decay Rate (n=13)	*P* value
Age, y			
Median (IQR)	29 (24-33)	31 (26-34)	0.828
Range	19-41	17-40	
Pre-ART CD4+ T-cell count, cells/μL			
Median (IQR)	267 (126-378)	363 (332-557)	0.192
Range	11-888	187-874	
Pre-ART CD8+ T-cell count, cells/μL			
Median (IQR)	775 (588-977)	1241 (626-2092)	0.294
Range	489-1460	436-2517	
Pre-ART CD4+/CD8+ T-cell count ratio			
Median (IQR)	0.34 (0.18-0.45)	0.35 (0.26-0.52)	0.447
Range	0.02-0.74	0.14-0.90	
Pre-ART plasma viremia, log10 copies/mL			
Median (IQR)	4.09 (3.61-4.38)	3.90 (3.41-4.43)	0.913
Range	2.50-4.91	2.73-4.62	
ART regimens, %			
2 NRTIs+1 INSTIs	2 (25.0)	1 (7.7)	0.646
2 NRTIs+1 NNRTIs	5 (62.5)	9 (69.2)	
2 NRTIs+1 PIs	1 (12.5)	3 (23.1)	

IQR, interquartile range; ART, antiretroviral therapy; NRTIs, nucleoside reverse transcriptase inhibitors; INSTIs, integrase strand transfer inhibitors; NNRTIs, non-nucleoside reverse transcriptase inhibitors; PIs, protease inhibitors.
